# JNK Activation Turns on LPS- and Gram-Negative Bacteria-Induced NADPH Oxidase-Dependent Suicidal NETosis

**DOI:** 10.1038/s41598-017-03257-z

**Published:** 2017-06-13

**Authors:** Meraj A. Khan, Armin Farahvash, David N. Douda, Johann-Christoph Licht, Hartmut Grasemann, Neil Sweezey, Nades Palaniyar

**Affiliations:** 1 Program in Translational Medicine, Peter Gilgan Centre for Research and Learning, The Hospital for Sick Children, Toronto, ON Canada; 2 Department of Laboratory Medicine and Pathobiology, University of Toronto, Toronto, ON Canada; 3 Departments of Paediatrics and Physiology, University of Toronto, Toronto, ON Canada; 4 Institute of Medical Sciences, Faculty of Medicine, University of Toronto, Toronto, ON Canada

## Abstract

Neutrophils cast neutrophil extracellular traps (NETs) to ensnare microbial pathogens. Nevertheless, the molecular rheostats that regulate NETosis in response to bacteria are not clearly established. We hypothesized that stress-activated protein kinase or c-Jun N-terminal Kinase (SAPK/JNK) is a molecular switch that turns on NETosis in response to increasing concentrations of lipopolysaccharide (LPS)- and Gram-negative bacteria. Here we show that *Escherichia coli* LPS (0111:B4; 10–25 μg/ml), but not phorbol myristate acetate (PMA), activates JNK in human neutrophils in a dose-dependent manner. JNK inhibitors SP600125 and TCSJNK6o, and a TLR4 inhibitor TAK242 suppress reactive oxygen species production and NETosis in LPS-, but not PMA-treated neutrophils. Diphenyleneiodonium suppresses LPS-induced NETosis, confirming that endotoxin induces NADPH oxidase-dependent NETosis. Immunoblots, Sytox Green assays, and confocal microscopy of cleaved caspase-3 and nuclear morphology show that JNK inhibition does not induce apoptosis in LPS-stimulated neutrophils. JNK inhibition also suppresses NETosis induced by two typical Gram-negative bacteria, *E. coli* and *Pseudomonas aeruginosa*. Therefore, we propose that neutrophils use a TLR4-dependent, JNK-mediated molecular sensing mechanism to initiate NADPH oxidase-dependent suicidal NETosis in response to increasing concentrations of LPS, and Gram-negative bacteria. The LPS-TLR4-JNK activation axis determines the fate of these cells: to be or not to be NETotic neutrophils.

## Introduction

Neutrophils release NETs, that are decondensed chromatin decorated with antimicrobial proteins^1, 2^. As cytotoxic protein-DNA complexes, NETs are beneficial antibacterial defense structures. However, dysregulated NETosis leads to the development and exacerbation of many autoimmune and chronic infectious and inflammatory diseases^3^. Despite the importance of NETosis regulation, the signaling pathways and molecular rheostats that regulate NETosis in response to bacteria are not fully understood. Nevertheless, it is well established that several Gram-negative bacteria activate NADPH oxidase 2 (Nox), which subsequently generates ROS to induce NETosis^2, 4–6^. This type of neutrophil death is called suicidal Nox-dependent NETosis^7, 8^. We and others have shown that several kinases are important for regulating Nox-dependent NETosis^7, 9^.

Previous studies show that MAPKs such as ERK and p38 regulate Nox-dependent NETosis^7, 8, 10, 11^. However, the role of JNK/SAPK in typical Nox-dependent NETosis signaling is not established. Our recent transcriptomics studies predict that the phorbol myristate acetate (PMA), the prototypic diacylglycerol (DAG) mimetic that induces Nox-dependent NETosis, does not induce JNK activation^12^. Low levels of LPS (e.g., 100 ng/ml) do not activate JNK unless the cells are primed with cytokines^13–15^; however, whether LPS at higher concentrations activates JNK or regulates NETosis induced by LPS-containing bacteria is unknown. In human neutrophils, JNK exists as two isoforms (46 kDa JNK1 and 54 kDa JNK2)^16^. The JNK/SAPK pathway becomes activated in response to various external stresses, and is involved in both pro- and anti-apoptotic responses depending on the cell types and pathways^17–19^. Here we tested whether JNK is an important molecular sensor that initiates NETosis in response to increasing concentrations of LPS- and Gram-negative bacteria.

PMA is used often as an agonist to induce Nox-dependent NETosis. It activates protein kinase C (PKC), which then activates Nox for producing ROS^7, 8, 10^. Although LPS induces NETosis, the mode of LPS-induced NETosis has not been completely resolved. The main concept has been that LPS engages TLR4 present on platelets, and subsequently the activated platelets directly or indirectly induce Nox-independent vital NETosis; however, certain types of LPS can induce Nox-dependent NETosis^20–23^. To determine the direct and regulatory effects of LPS on NETosis, we induced NETosis with PMA, LPS and two typical Gram-negative bacteria, *E. coli* and *P. aeruginosa*. For the first time, we found that LPS, but not PMA, activates JNK in a dose-dependent fashion, and that JNK activation is responsible for LPS-, but not PMA-, mediated ROS production and subsequent Nox-dependent suicidal NETosis. Furthermore, during LPS-mediated stimulation of neutrophils, inhibition of TLR4 signaling by TAK242 or JNK activity by SP600125 and TCSJNK6o suppress NETosis. JNK inhibition does not lead to apoptosis or other forms of cell death; instead, it maintains the viability of neutrophils. Gram-negative bacteria such as *E. coli* and *P. aeruginosa* also induce NETosis in a multiplicity of infection (MOI)-dependent manner that can be significantly inhibited by SP600125. Therefore, JNK acts as a molecular rheostat that uniquely regulates LPS-mediated NETosis by regulating ROS production in neutrophils.

## Results

### LPS treatment induces JNK activation in human neutrophils

**Figure 1:**
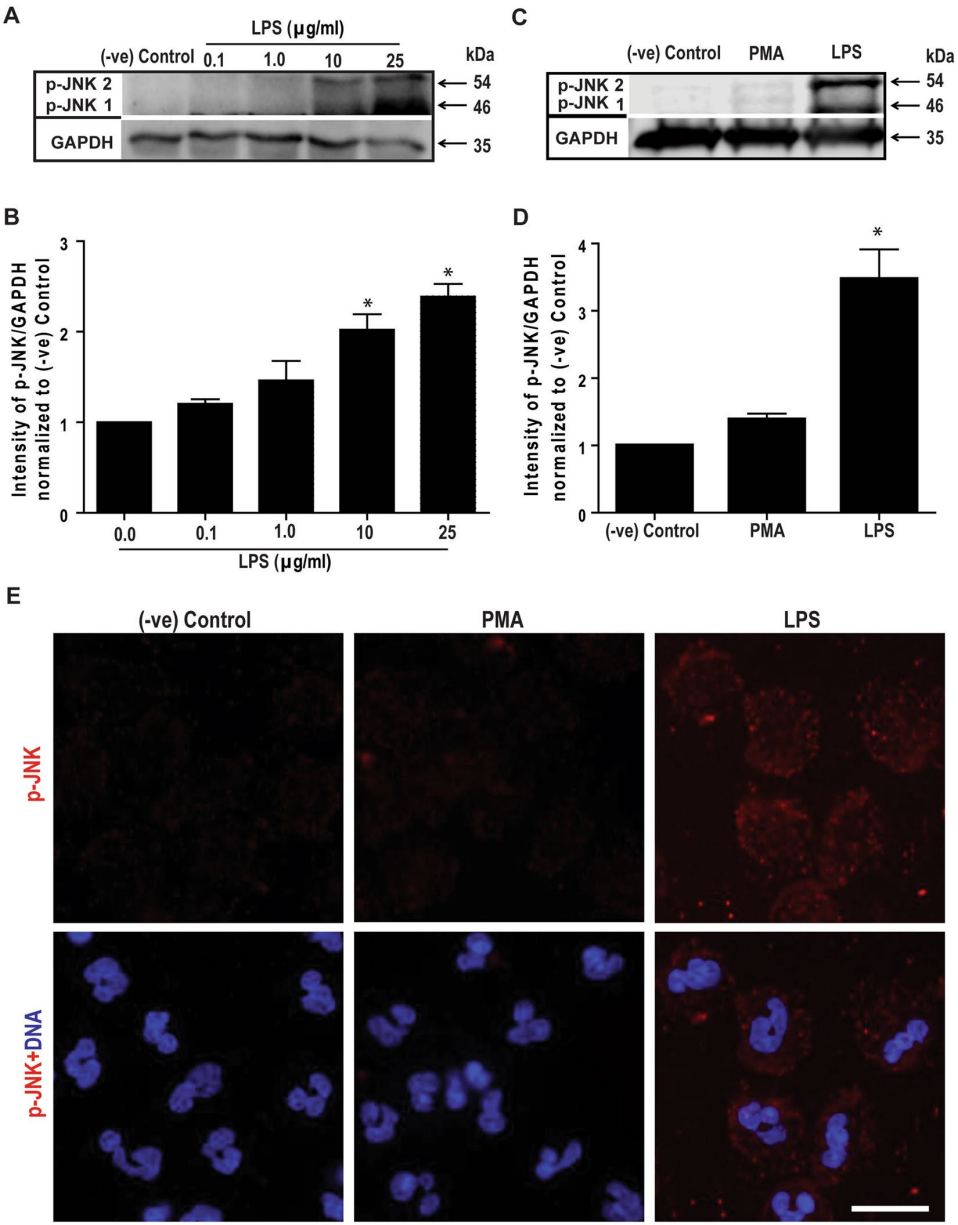
**LPS, but not PMA, dose-dependently activates JNK in human neutrophils**. (**A**) Human neutrophils were stimulated with *E. coli* LPS (0111:B4; 0, 0.1, 1.0, 10, 25 μg/ml) for 30 min, and lyzed for Western blot analyses. Immunoblots show an LPS dose-dependent phosphorylation of JNK (p-JNK). GAPDH blots were used as loading controls (n = 3). (**B**) The densitometry analyses show the significant dose-dependent increase of the JNK activation in LPS (10 and 25 μg/ml) treated neutrophils. The values were normalized to the negative control values of the same experiment (*indicates p-value < 0.05; One-sample t test compare to hypothetical value 1). (**C**) Human neutrophils were stimulated with media (-ve control), PMA (25 nM) or LPS (25 μg/ml) for 30 minutes. Immunoblots show that LPS, but not PMA activates JNK in neutrophils. GAPDH blots were used as loading controls (n = 5). (**D**) The densitometry and statistical analyses were conducted for **C**, as of **B**. Total JNK1 and JNK 2 levels do not change within 30-minute incubation period (Supplementary Fig. S1). Error bars in all the panels represent SEM. (**E**) Confocal microscopy images of the neutrophils immunostained with p-JNK (red), and DNA (blue) after 30 min of NETosis induction, confirm the increased activation of JNK in LPS-, but not PMA-mediated NETosis (n = 3; scale bar 25 μm). See the full Western blots in Supplementary Fig. S1.

To determine the relevance of JNK in NETosis, we examined the effect of LPS on JNK activation in neutrophils. Western blot analyses show that incubating neutrophils with different concentrations of *E. coli* LPS (0111:B4; 0–25 μg/ml) for 30 minutes phosphorylates JNK (p-JNK) to different levels. At baseline, phosphorylation of both JNK1 and JNK2 is hardly detectable, but activation increases with increasing concentrations of LPS (Fig. 1A, B). At 100 ng/ml LPS, which is a concentration routinely used for studying neutrophil activation and degranulation, JNK activation is very low (almost at baseline). At 1 μg/ml LPS, phosphorylation levels are highly variable. However, at 10 and 25 μg/ml LPS, JNK activation is consistently higher than the baseline and other lower concentrations of LPS.

Comparing LPS (25 μg/ml) with the prototypic Nox-dependent NETosis agonist PMA (25 nM) shows that JNK is highly activated in LPS-treated cells while no activation above baseline is detected in neutrophils incubated with PMA (Fig. 1C–D). The expression of total JNK1 and JNK2 (t-JNK) does not change within a 30-minute incubation period. Similar levels of t-JNK are present in resting neutrophils, and neutrophils treated with PMA and LPS (Supplementary Fig. S1). Therefore, the increase in p-JNK is directly attributable to the phosphorylation of existing JNK, rather than to new protein synthesis. Confocal microscopy images of the neutrophils stained for DNA (DAPI, blue) and immunostained for p-JNK (red) confirm the activation of JNK in neutrophils treated with LPS (Fig. 1E). Therefore, LPS, but not PMA, activates JNK in neutrophils in a dose-dependent manner; consistent and substantial levels of JNK activation are detected only at higher LPS concentrations (10–25 μg/ml).

### Inhibition of JNK activation and TLR4 signaling suppress LPS-induced ROS production

**Figure 2:**
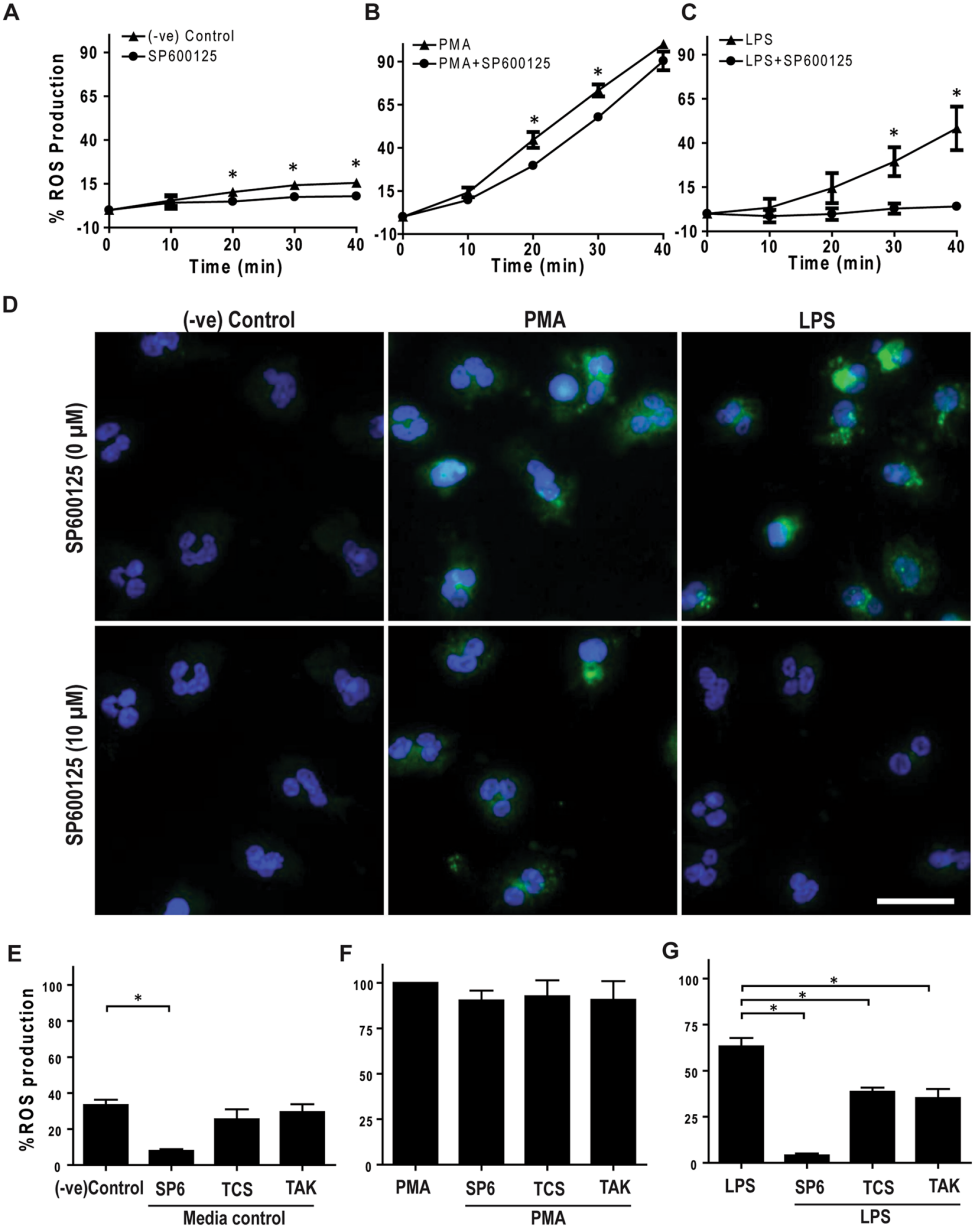
**LPS-mediated ROS production in human neutrophils depends on TLR4 signaling and JNK activation**. Human neutrophils were treated with cytosolic ROS indicator dye DHR123 and activated with PMA (25 nM), LPS (25 μg/ml) or only media (-ve control) in the presence or absence of JNK inhibitor SP600125 or TCSJNK6o (TCS), or TLR4-TIRAP/TRAM inhibitor TAK242 (TAK). (**A–C; E–G**) R123 based ROS generation kinetics by plate reader assays show that both PMA and LPS induce ROS generation, over 40 min of post activation. JNK inhibition with both SP600125 and TCSJNK6o drastically reduces the generation of ROS in LPS treated Neutrophils, while inhibitors do not suppress ROS generation in neutrophils activated with PMA. The ROS values were calculated by considering the PMA-mediated ROS production as 100% at 40-minute time point (n = 3, *p-value < 0.05; Two-way ANOVA with Bonferroni’s multiple comparison post test). (**D**) Confirmation of generation and inhibition of ROS by confocal imaging. R123 (green) and DNA (blue). Confocal images confirm the inhibition of ROS in LPS activated neutrophils but not in neutrophils activated with PMA, at 60-minute time point (n = 3; scale bar 20 μm). (**E–G**) Measuring R123-based ROS generation kinetics by plate reader assays show that TLR4-TIRAP/TRAM inhibition with TAK242 significantly reduces the generation of ROS in LPS treated Neutrophils. However, the inhibitor does not suppress ROS generation in neutrophils activated with PMA. The ROS values were calculated by considering the PMA-mediated ROS production as 100%, at 40-minute time point (n = 3, *p-value < 0.05; One-way ANOVA with Tukey’s post test compared to negative control). See Supplementary Fig. S2 for relative changes in ROS production. Error bars in all the panels represent SEM.

Since both PMA and LPS can induce ROS production, we next examined the effects of JNK inhibition in ROS production. DHR123 is a non-fluorescent dye. Upon binding to intracellular ROS, DHR123 is converted to R123, which emits a green fluorescence signal^24^. SP600125 is a commonly used JNK inhibitor^13, 25–27^; hence, we performed the DHR123 assay to determine the amount of ROS production following PMA or LPS treatment, in the presence or absence of 10 μM SP600125. Plate reader assays show that the presence of SP600125 suppresses background ROS production in the media control. SP600125 only slightly suppresses PMA-mediated ROS production (Fig. 2A–B). By contrast, the presence of SP600125 strongly suppresses LPS-mediated ROS production (Fig. 2C). Images of the neutrophils confirm the strong suppression of ROS production by the JNK inhibitor in LPS-, but not in PMA-, treated cells (Fig. 2D). These data show that JNK inhibitor SP600125 suppresses LPS-, but not PMA-mediated ROS production in neutrophils.

Although SP600125 suppresses the activation of all isoforms of JNK, some of its effects occur indirectly via suppressing other kinases^26, 28, 29^. Therefore, to further validate the role of JNK activation in ROS production, we used another JNK inhibitor TCSJNK6o, which shows >1000-fold specificity to JNK over other kinases^30^. When the ROS experiments were repeated in the presence or absence of 10 μM TCSJNK6o, plate reader assays show that the inhibitor significantly suppresses LPS-, but not PMA-, mediated ROS production (Fig. 2E–G; Supplementary Fig. S2A–C). Both of these JNK inhibitors suppressed LPS-mediated ROS production. Therefore, inhibition of JNK suppresses LPS-, but not PMA-, mediated ROS production in neutrophils.

Neutrophils express TLR4 and appropriate signaling molecules, and LPS exerts its intracellular signaling via TLR4 and MyD88-like adaptor proteins^31, 32^. Therefore, to determine whether the LPS-mediated ROS production is dependent on TLR4 signaling, in ROS assays we used an inhibitor (TAK242) that specifically blocks the interaction between TLR4 and its intracellular adaptor proteins^33–35^. This inhibitor also significantly suppresses ROS production in neutrophils treated with LPS, but not PMA (Fig. 2E–G; Supplementary Fig. S2A–C). Therefore, both JNK activation and TLR4 signaling are important for LPS-mediated ROS production in neutrophils.

### Inhibition of JNK activation and TLR4 signaling suppress LPS-mediated NETosis

**Figure 3:**
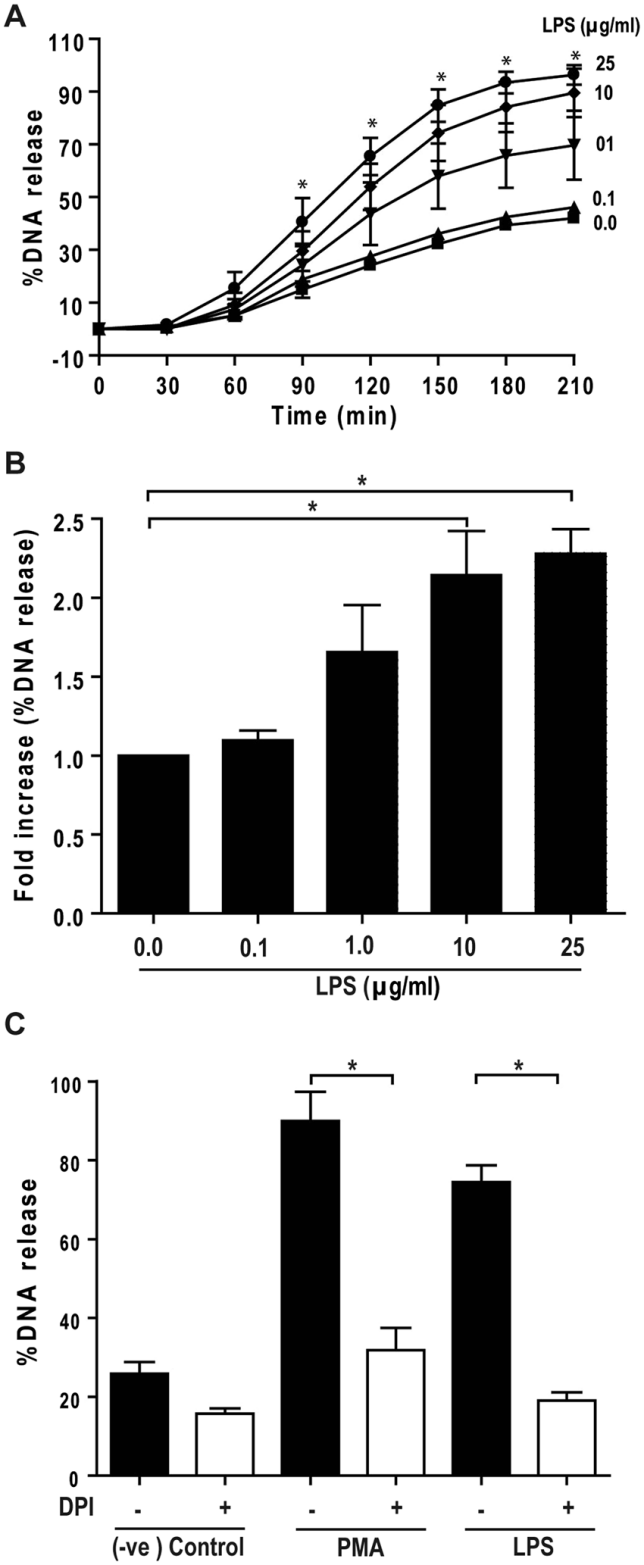
**LPS dose-dependently induce Nox-dependent NETosis, and JNK activation is upstream of Nox activation**. (**A, B**) LPS dose (0, 0.1, 1, 10, 25 μg/ml)-dependent NETosis kinetics was assessed by Sytox Green plate reader assays. (**A**) Considerable NETosis is not detected in the presence of lower concentrations of LPS (e.g., 100 ng/ml). At 1 μg/ml LPS concentration, NETosis is highly variable. Substantial amount of NETosis is detectable in the presence of higher concentrations of (10–25 μg/ml) LPS. %DNA release (NETosis) at the last time point with 25 μg/ml LPS is considered as 100% (n = 3; Two-way ANOVA with Bonferroni’s multiple comparison post test). (**B**) Fold differences in NETosis was calculated from Panel A (n = 3; One-sample t test compare to hypothetical value 1). (**C**) Sytox Green plate reader assays show that Nox inhibitor DPI suppresses LPS-mediated NETosis. Inhibition of PMA-mediated NETosis confirms that DPI inhibits Nox-dependent NETosis (n = 3; One-way ANOVA with Tukey’s multiple comparison post test). See Supplementary Fig. S3 for the kinetics of the dose dependent response of DPI during LPS-mediated NETosis. Error bars in all the panels represent SEM.

Next, we used the common Sytox Green fluorescence plate reader assay as a proxy to investigate the role of JNK in NETosis. Sytox Green is a cell impermeable dye, and emits green fluorescence upon intercalating with the DNA released during NETosis. These assays show that increasing concentrations of LPS increases NETosis (Fig. 3A). At 100 ng/ml, LPS does not induce NETosis. As indicated by the large error bars, induction of NETosis is highly variable at an LPS concentration of 1 μg/ml. In certain experiments, LPS at 1 μg/ml does not induce NETosis, whereas in other experiments, LPS at this concentration induces substantial levels of NETosis. By contrast, 10–25 μg/ml of LPS reproducibly induces NETosis, resulting in >2.5 fold DNA release compared to baseline conditions. Therefore, LPS concentration is a key factor that determines whether neutrophils undergo NETosis or not.

Various agonists induce different types of NETosis: Nox-dependent or -independent NETosis^3, 10, 36^. To determine the type of NETosis that LPS induces, we conducted the experiments in the presence or absence of diphenyleneiodonium (DPI), an inhibitor of Nox. Sytox Green assays show that DPI not only inhibits PMA-, but also suppresses LPS (25 μg/ml)-mediated NETosis in a dose-dependent manner (Fig. 3C; Supplementary Fig. S3). Therefore, the *E. coli* endotoxin induces suicidal Nox-dependent NETosis.

**Figure 4:**
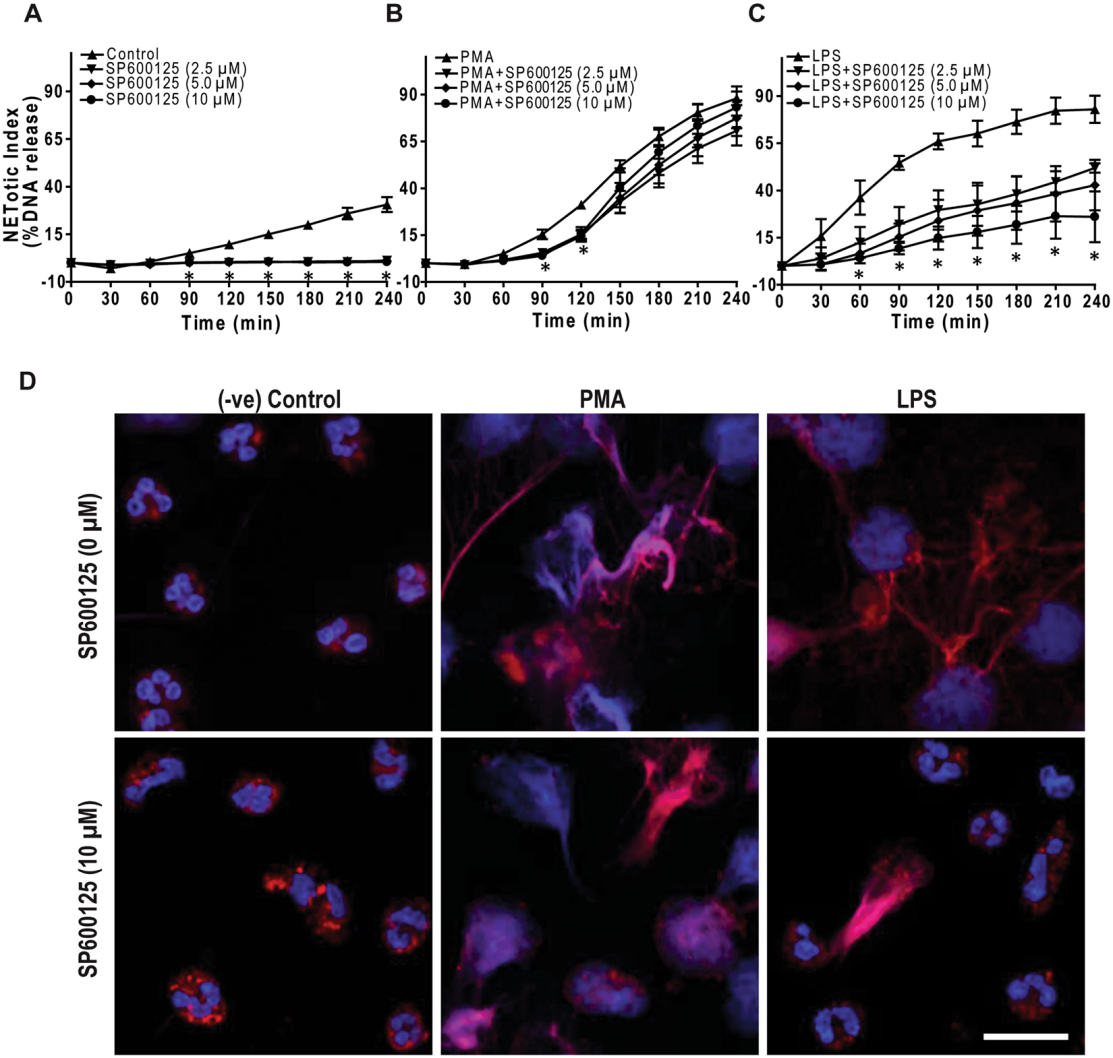
**JNK inhibition by SP600125 suppresses LPS-mediated NETosis**. (**A–C**) NETosis kinetics was assessed by Sytox Green plate reader assay after activation with 25 nM PMA and 25 μg/ml LPS in the presence or absence of inhibitor. As shown in the %DNA release analysis, SP600125 (2.5, 5.0, 10 μM) suppresses LPS mediated NETosis in a dosage-dependent manner, while not in PMA mediated NETosis (n = 3–4; *p value < 0.05; Two-way ANOVA with Bonferroni’s post test conducted at each time point). Error bars in all the panels represent SEM. (**D**) Neutrophils were activated by PMA and LPS with and without SP600125 for 4 hours, immunostained, and imaged for myeloperoxidase (MPO) and DNA. MPO is visible around the nuclei in media control with or without SP600125. MPO co-localizes to NET DNA generated by LPS, PMA, and PMA with SP600125. Neutrophils treated with LPS and SP600125 do not show NETosis, and the nuclear morphology of these cells remains the same as that of the unstimulated control neutrophils (Blue, DAPI staining for DNA; Red, MPO; n = 3; scale bar 20 μm). See Supplementary Fig. S4 for low magnification images.

To determine the role of JNK in NETosis, we conducted the Sytox Green assay in the presence or absence of SP600125. The JNK inhibitor suppresses LPS-mediated NETosis in a dose-dependent manner, whereas the inhibitor does not substantially inhibit PMA-mediated NETosis (Fig. 4A–C). JNK inhibitor suppresses the baseline NETosis in media control, while it slightly suppresses PMA-mediated NETosis, at early time points. To confirm NETosis, neutrophils were treated with PMA or LPS (25 μg/ml), in the presence or absence of SP600125, for 4 hours and immunostained for myeloperoxidase (MPO). During NETosis, MPO enters nucleus and is found associated with chromatin. Presence of MPO on extracellular chromatin is considered to represent NET formation^2, 7, 8, 37^. Immunoconfocal microscopy images show that MPO is localized in the cytoplasm, around the nuclei, in negative controls either in the presence or absence of SP600125. By contrast, MPO co-localizes to NETs, which were induced by incubating neutrophils with LPS or PMA. SP600125 does not noticeably inhibit PMA-mediated NET formation. However, LPS does not induce NETosis in the presence of SP600125; the nuclear morphology of these cells remains similar to that of the unstimulated control neutrophils (Fig. 4D; Supplementary Fig. S4). Therefore, inhibiting JNK activation by SP600125 suppresses NETosis.

**Figure 5:**
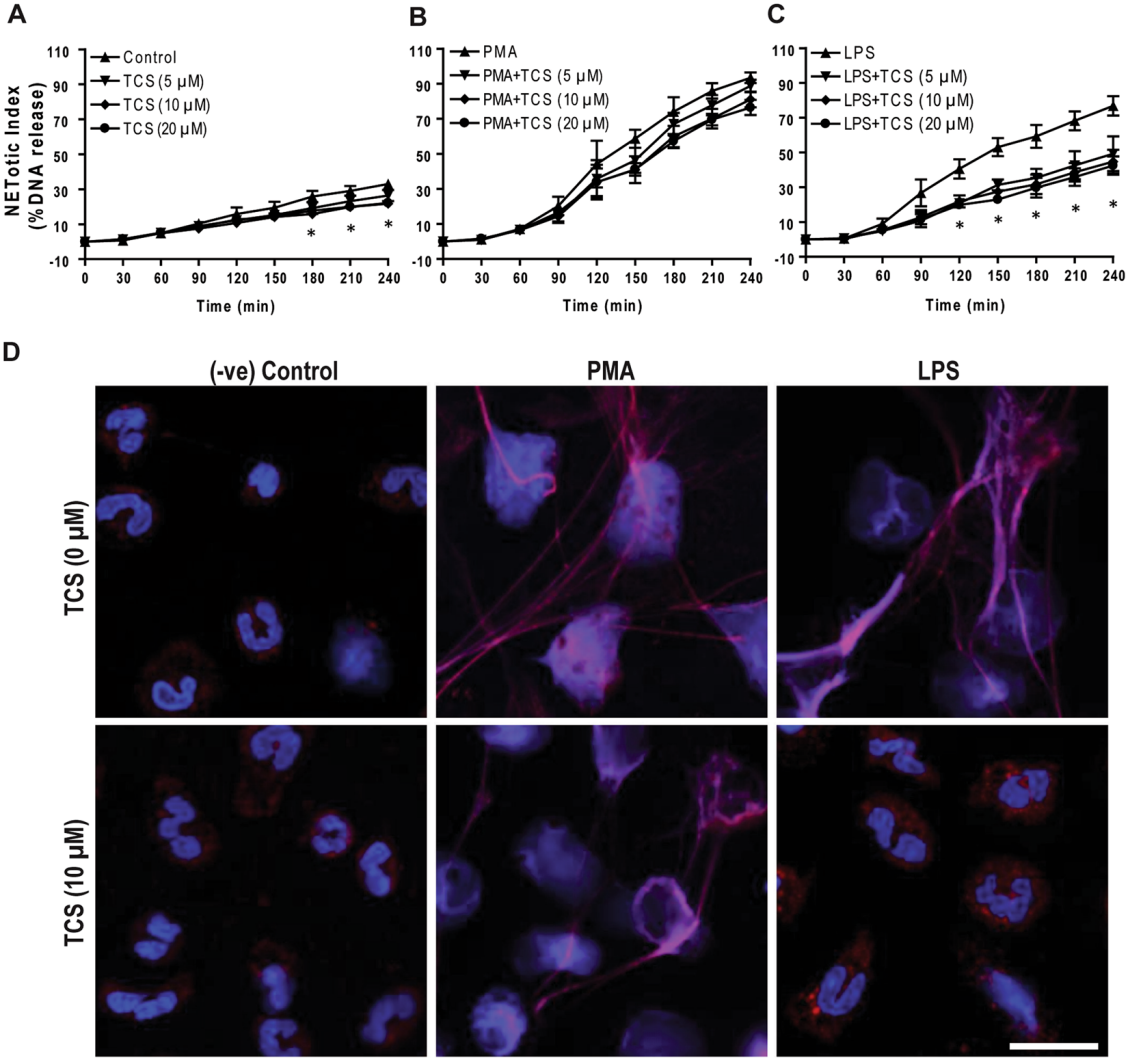
**JNK inhibition by TCSJNK6o suppresses LPS-mediated NETosis.** (**A–C**) NETosis kinetics was assessed by Sytox Green plate reader assay after activation with 25 nM PMA and 25 μg/ml LPS in the presence or absence of TCSJNK6o. As shown in the %DNA release analysis, TCSJNK6o (TCS; 5, 10, 20 μM) suppresses LPS mediated NETosis, while not in PMA mediated NETosis (n = 3–4; *p value < 0.05; Two-way ANOVA with Bonferroni’s post test conducted at each time point). Error bars in all the panels represent SEM. (**D**) Neutrophils were activated by PMA and LPS with and without TCSJNK6o for 4 hours, immunostained, and imaged for myeloperoxidase (MPO) and DNA. MPO is visible around the nuclei in media control with or without TCSJNK6o. MPO co-localizes to NET DNA generated by LPS, PMA, and PMA with TCSJNK6o. Treating neutrophils with LPS in the presence of TCSJNK6o does not result in NETosis, and the nuclear morphology of these cells remains the same as that of the unstimulated control neutrophils (Blue, DAPI staining for DNA; Red, MPO; n = 3; scale bar 20 μm). See Supplementary Fig. S5 for low magnification images.

We repeated the entire set of experiments with a second JNK inhibitor, TCSJNK6o. This inhibitor also suppresses baseline and LPS (25 μg/ml)-, but not PMA-, mediated NETosis as determined by Sytox Green (Fig. 5A–C) and confocal immunofluorescence microscopy (Fig. 5D; Supplementary Fig. S5). These data (Figs 4 and 5, Supplementary Figs S4 and S5) show that JNK activation is necessary for the induction of LPS-, but not PMA-mediated NETosis.

**Figure 6:**
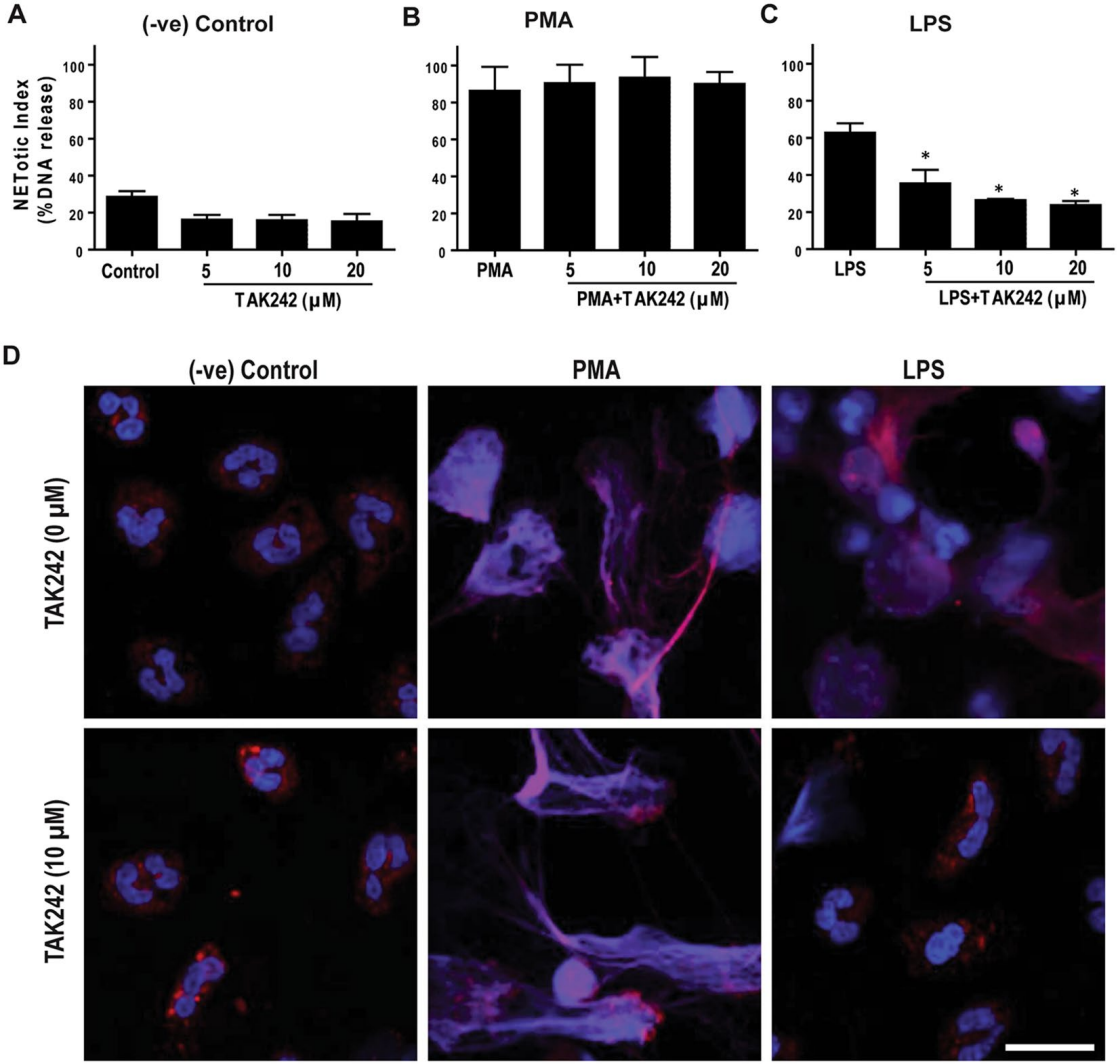
**TLR4 signaling inhibition by TAK242 suppresses LPS-mediated NETosis**. (**A–C**) NETosis kinetics was assessed by Sytox Green plate reader assay after activation with 25 nM PMA and 25 μg/ml LPS in the presence or absence of TAK242. As shown in the %DNA release analysis, TAK242 (5, 10, 20 μM) suppresses LPS mediated NETosis, while not in PMA mediated NETosis (n = 3–4; *p value < 0.05; One-way ANOVA with Tukey’s multiple comparison post test). Error bars in all the panels represent SEM. (**D**) Neutrophils were activated by PMA and LPS with and without TAK242 for 4 hours, immunostained, and imaged for myeloperoxidase (MPO) and DNA. MPO is visible around the nuclei in media control with or without TAK242. MPO co-localizes to NET DNA generated by LPS, PMA, and PMA with TAK242. Treating neutrophils with LPS in the presence of TAK242 does not results in NETosis, and the nuclear morphology of these cells remains the same as that of the unstimulated control neutrophils (Blue, DAPI staining for DNA; Red, MPO; n = 3; scale bar 20 μm). See Supplementary Fig. S6 for low magnification images.

To determine the role of TLR4 signaling in LPS-mediated NETosis, we incubated the neutrophils with different concentrations of TLR4 inhibitor TAK242, and induced NETosis with LPS (25 μg/ml) or PMA. Both Sytox Green assays (Fig. 6A–C) and confocal immunofluorescence microscopy (Fig. 6D; Supplementary Fig. S6) show that the TLR4 inhibitor suppresses LPS-, but not PMA-, mediated NETosis. These studies show that TLR4 signaling is important for LPS-mediated NETosis.

### JNK inhibition suppresses death in neutrophils treated with LPS

**Figure 7:**
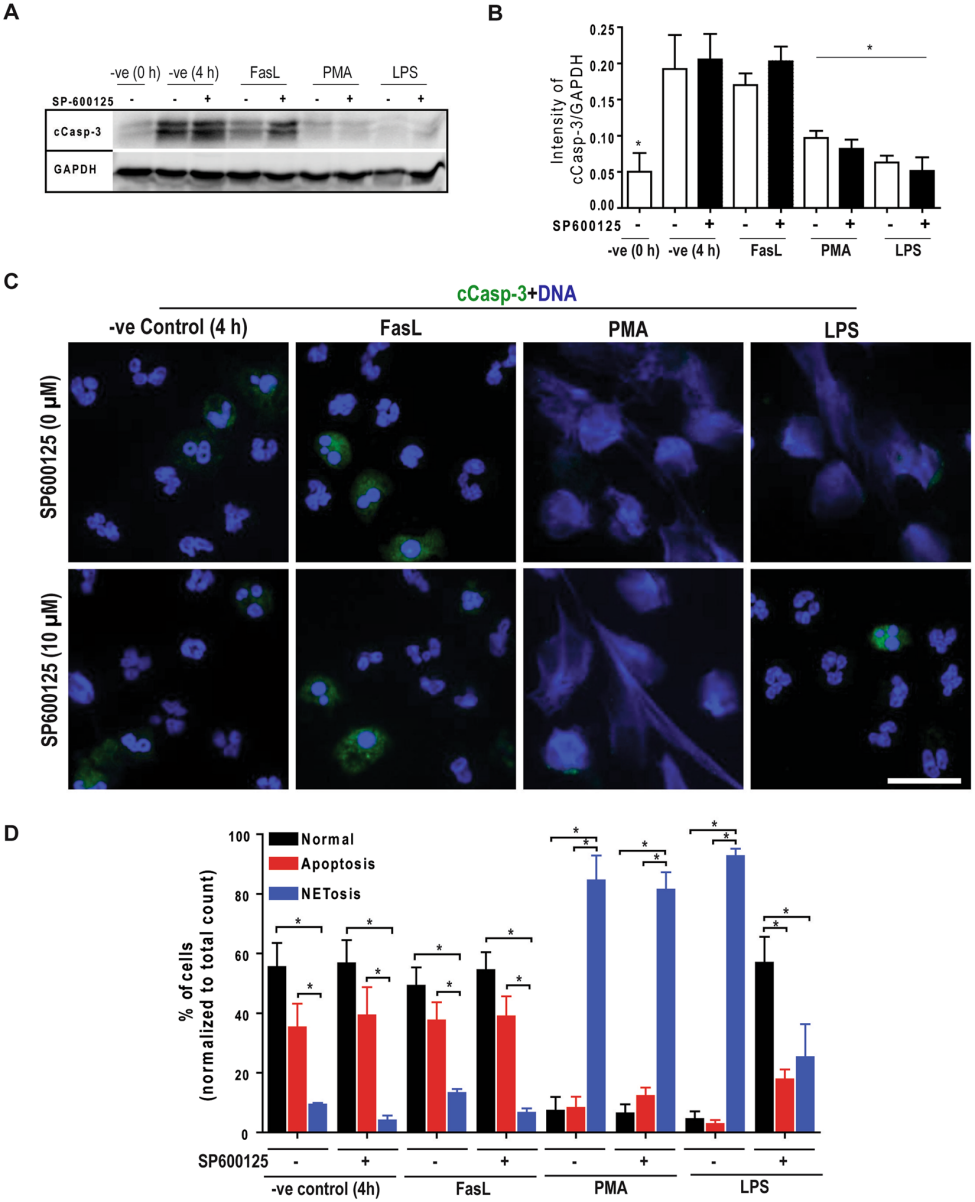
Inhibition of JNK in LPS-treated neutrophils does not lead to apoptosis, and maintains cell survival. (**A–B**) Immunoblot analyses of the cleave caspase-3 (cCasp-3) show that neutrophils treated with media control for 4 hours in the presence or absence of SP600125 undergo apoptosis to the extent similar to that of Fas-L treatment. Neutrophils activated by 25 nM PMA and 25 μg/ml LPS mostly show NETosis. Treating neutrophils with LPS and SP600125 shows no cCasp-3; NETosis in these cells are very low (*p < 0.05; One-way ANOVA with Tukey’s multiple comparison post test). (**C**) Confocal microscopy of the neutrophils stained with DAPI (blue) and cCasp-3 (green) after 4 hours. Images show some of the non-activated control neutrophils stain for cCasp-3, in the presence or absence of SP600125. Treating neutrophils with LPS, PMA and PMA with SP600125 exclusively show signs of NETosis; only traces of cCasp-3 is visible in a few of these cells. Treating neutrophils with LPS and SP600125 show no cCasp-3 or NETs (n = 3; scale bar 20 μm). See Supplementary Fig. S7 for the low magnification images. (**D**) The percentages of normal, apoptotic and NETotic cells were calculated based on cCasp3 staining and nuclear morphology. The quantitative analysis confirms the qualitative analysis (n = 3; *p < 0.05; One-way ANOVA with Tukey’s multiple comparison post test). Error bars in all the panels represent SEM. Collectively, inhibition of JNK activation during LPS-mediated activation of neutrophils result is cell survival.

To determine the effect of JNK inhibition during LPS-mediated activation of neutrophils, we next examined the fate of neutrophils stimulated with LPS (25 μg/ml) or PMA. We performed immunoblot analyses of cleaved caspase-3 in control neutrophils, and in neutrophils incubated with PMA or LPS, in the presence or absence of JNK inhibitor SP600125. The immunoblots show that freshly isolated neutrophils have barely detectable cleaved caspase-3; the neutrophils incubated for 4 hours underwent apoptosis to an extent similar to FasL-treated cells. In repeated experiments, the presence or absence of SP600125 does not make any significant difference in the degree of apoptosis in the control cells. By contrast, neutrophils activated with PMA or LPS do not show a significant degree of caspase-3 activation compared to controls (Fig. 7A, B).

We also conducted fluorescence microscopy after immunostaining for cleaved caspase-3 (cCasp-3; green) to assess NETosis and apoptosis by nuclear morphology and caspase-3 cleavage in these cells. In the control neutrophils, the presence of JNK inhibitor does not change the nuclear morphology (Fig. 7C; Supplementary Fig. S7). The 4-hour media control and FasL conditions show cleavage of caspase-3 regardless of the presence of SP600125. By contrast, NET induction with PMA or LPS does not result in caspase-3 activation at 4 hours, regardless of the presence of the JNK inhibitor (Fig. 7C). These images confirm the Western blot data. Manually quantifying the number of cells undergoing different types of cell death shows that JNK inhibition in LPS-treated neutrophils suppresses apoptosis in these cells (Fig. 7D). We did not observe any other caspase 3-independent death, based on cCasp-3 staining and nuclear morphology. Collectively, this data set (Fig. 7; Supplemental Fig. S7; Sytox Green data in Figs 4–6 that show no increase in cell permeability in the presence of the inhibitor) shows that inhibition of JNK during NETosis does not redirect NETosis to apoptosis; instead, JNK inhibition maintains the viability of neutrophils stimulated with LPS.

### JNK inhibition suppresses *E. coli*- and *P. aeruginosa*-induced NETosis

**Figure 8:**
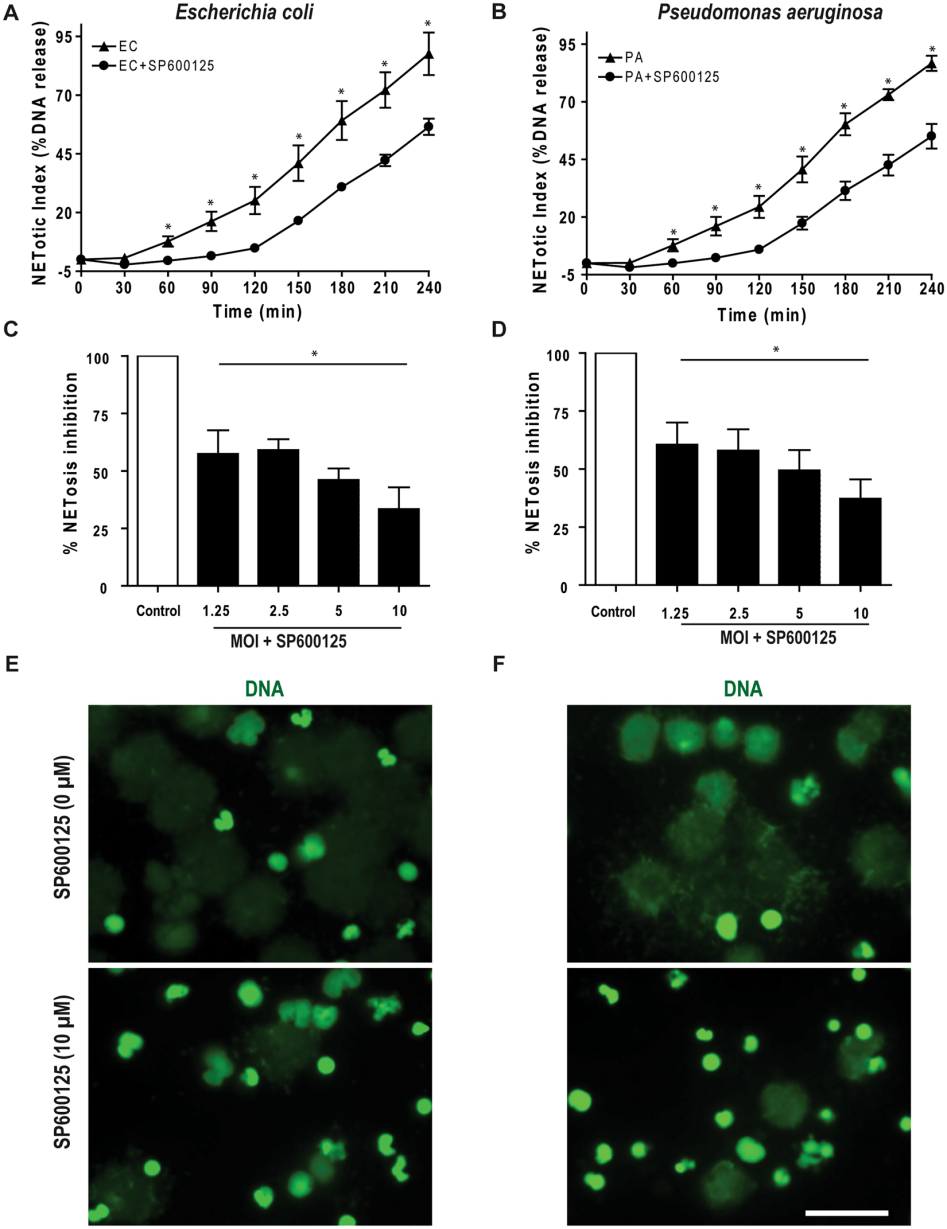
JNK inhibition suppresses both *Escherichia coli-* and *Pseudomonas aeruginosa-*mediated NETosis. (**A-B**) Sytox Green assays show the induction of NETosis by both *E. coli* (EC) and *P. aeruginosa* (PA; n = 3; *p value < 0.05; t-test). (**C–D**) Pretreatment of neutrophils with SP600125 and subsequent activation by *E. coli* and *P. aeruginosa* (0, 1.25, 2.5, 5.0, 10 MOI each), show significant increase in NETosis inhibition in each MOIs (n = 3; *p value < 0.05; One-way ANOVA with Dunnett’s post test). The NETosis induced by each bacterium in the absence of SP600125 was considered as 100%. Error bars in all the panels represent SEM. (**E–F**) Confocal images captured on the same day after completing the 4 hour NETosis assays, show clear NETs DNA (green) in *E. coli* and *P. aeruginosa* induced neutrophils along with NETosis inhibition in case of SP600125 pretreated conditions (n = 3; scale bar 25 μm).

To demonstrate additional biological relevance of the JNK involvement in NETosis signaling, we studied the role of JNK in Gram-negative bacteria-induced NETosis. NETosis determined by Sytox Green assays and imaging show that both *E. coli* and *P. aeruginosa* induce NETosis. We found that JNK inhibitor SP600125 significantly suppresses NETosis induced by both of these bacteria (Fig. 8A,B). The degree of JNK-mediated NETosis suppression is higher when the number of bacteria are lower (Fig. 8C,D). NET images also indicate that JNK inhibitor suppresses bacteria-induced NETosis (Fig. 8E,F). These findings (Fig. 8) show that JNK also regulates NETosis induced by Gram-negative bacteria.

## Discussion

Previous studies have attempted to identify the role of JNK in neutrophil functions^18, 19, 26, 38–40^. However, the results were not conclusive, and the involvement of this stress-activated protein kinase (JNK/SAPK) in NETosis was not clearly established. In attempts to identify the functions of JNK in NETosis, we found that JNK activation is barely detectable in media control and PMA-treated neutrophils. In some experiments, PMA showed a slight, inconsistent activation of JNK above background, but considering all the biological replicates, the activation is not significantly different than non-activated neutrophil controls. The absence of JNK phosphorylation during the activation of neutrophils by PMA is consistent with our recent transcriptomics studies, which predict that PMA does not activate JNK^12^. By contrast, high levels of JNK activation occurs in response to increasing concentrations of LPS and a substantial level of JNK phosphorylation is detectable at higher LPS concentrations (Fig. 1; Supplementary Fig. S1). This LPS-mediated JNK activation is consistent with one previous report, but not with others^13, 38^. These differences could be due to the amount and types of LPS used in different studies. Inflammatory cytokines may also potentiate cells to activate JNK at low concentrations of LPS^15, 38^. Some of these reports also relied on indirect measures of JNK activation, whereas we directly determined JNK activation by Western blots. Based on our direct data, it is clear that LPS induces JNK activation in neutrophils in a dose-dependent fashion.

It has previously been established that both PMA and LPS induce ROS production and NETosis^6, 7, 35, 37, 41, 42^. Interestingly, inhibiting JNK activation leads to suppression of ROS production in LPS-, but not in PMA-treated neutrophils (Fig. 2; Supplementary Fig. S2). SP600125 and TCSJNK6o treatments suppress baseline ROS production in negative control cells, and these inhibitors slightly reduce ROS production in the presence of PMA at initial time points. This slight difference in ROS production is attributable to JNK-mediated baseline ROS production. By contrast, ROS production in LPS-treated cells relies fully on JNK activation. Cells including neutrophils respond to LPS via TLR4 signaling^31, 32, 43^. TAK242 specifically blocks the interaction between TLR4 and its intracellular adaptor proteins TIRAP (Toll/interleukin-1 receptor (TIR) domain-containing adaptor protein; also known as MyD88-adapter-like protein or Mal) and TRAM (TIR-domain-containing adapter-inducing interferon-β (TRIF)-related adaptor molecule^33–35^. Blocking TLR4 interaction with TIRAP/TRAM with TAK242 suppresses LPS-mediated ROS production (Fig. 2). LPS does not utilize MyD88-independent signaling mediated via TRAM^31, 32^, hence the TIRAP branch of the TLR4 signaling pathway must regulate ROS production. Furthermore, TLR4-TIRAP signaling can activate MAPK cascade such as MAPK kinase (MKK) 4 and 7 that subsequently activate JNK^44^. Therefore, *E. coli* (0111:B4) LPS likely regulates ROS production via TLR4-TIRAP-JNK signaling.

PMA exerts its effect by PKC-mediated activation of Nox, ROS production and subsequent NETosis^9, 45^. Inhibition of ROS generated by Nox (e.g., DPI) leads to the suppression of Nox-dependent NETosis^7, 9, 45^. We report here that DPI blocks LPS-mediated NETosis (Fig. 3; Supplementary Fig. S3). Therefore, JNK activation is an upstream event, and that LPS-mediated TLR4 signaling is responsible for JNK activation, which is important for Nox-dependent ROS production and NETosis. JNK can activate transcription factors and promote transcription to help decondense chromatin because our previous studies show that LPS-mediated NETosis could be suppressed by the transcription initiation inhibitor Actinomycin D^12^. Since transcription is a near terminal event in the NETosis pathway, Actinomycin D will inhibit NETosis regardless of the upstream events. The fact that JNK inhibition blocks Nox-mediated ROS production indicates that JNK also plays an upsteam role in activating Nox during LPS-mediated NETosis.

Low concentrations of LPS increase the lifespan of neutrophils^46^, whereas high concentrations of this Gram-negative bacterial cell wall component induces NETosis^6^. Here we show that inhibition of JNK suppresses LPS-induced, but not PMA-mediated NETosis in a dose-dependent manner (Figs 1–5). Inhibition of JNK also reduced the background NETosis in the media control reflecting the relevance of background ROS activation in media control for NETosis (Figs 4 and 5). The same mechanism may be attributable to the slight decrease in PMA-induced NETosis at early time points, when treated with JNK inhibitors. Moreover, immunohistochemistry confirms that JNK is essential to LPS-induced NETosis. MPO is stored in cytoplasmic azuropilic granules in intact neutrophils, but during NETosis it is found on the chromatin^2, 7, 37^. The majority of nuclei are intact and MPO is found in cytoplasmic granules in control cells, unaffected by inhibition of JNK (Figs 4 and 5) or TLR4 (Fig. 6). Similar to the PMA treatment, LPS induces chromatin decondensation and NET release along with MPO. These results confirm NETosis^2, 7, 37^. However, JNK inhibition suppresses LPS-induced NETosis such that the nuclear morphology resembles the media control and the MPO granules are in the cytoplasm. This remarkable finding demonstrates that inhibiting JNK activation suppresses LPS-mediated ROS production and maintains neutrophils similar to live cells even in the presence of NETosis-inducing concentrations (10–25 μg/ml) of LPS.

A recent study indicates that LPS from different bacteria induce NETosis to different degrees, and 8 pg LPS (0111:B4) per neutrophil does not induce NETosis on its own^20^. However, the same amount of LPS (0111:B4) robustly induce Nox-independent NETosis in the presence of platelets^20^. Previous studies also used a similar range of concentrations of LPS (0111:B4; e.g., 0.1–5 μg/ml) and identified that the interaction between LPS and the TLR4 present on platelets effectively induces NETosis^21, 22^. Our studies show that robust and reproducible levels of JNK activation and NETosis are detected in the LPS range of 10–25 μg/ml, which is equivalent to 20–50 pg LPS per neutrophil (100 μl of 10–25 μg/ml LPS for 50,000 neutrophils); thus, the amount of LPS is a key factor for inducing JNK activation, and subsequent Nox-dependent NETosis (Figs 1–6; Supplementary Figs 1–6).

Biological systems are highly regulated to avoid unnecessary harm to the host. Although platelets are present together with neutrophils in the blood and in the wounds with damaged capillaries, both of these cell types are not present in all infection sites. The induction of vital Nox-independent NETosis due to platelet-neutrophil interactions (LPS:TLR4:platelet:neutrophil) described by others^20–22^ may be relevant to clot formation with no ROS production or intravascular NETosis. Furthermore, high blood concentrations of LPS will induce sepsis and multiorgan failure, which are not consistent with supporting the life of the host^47, 48^. By contrast, neutrophils, but not platelets, have the capacity to migrate into various tissues or sites of infection and inflammation^49, 50^. In these loci, neutrophils need to sense the bacterial load and initiate NETosis only when the bacterial load is high. Indeed, local LPS concentrations can be very high at the site of bacterial infection. Hence, we propose that LPS concentration is a key factor that neutrophils use for directly sensing the bacterial load in these milieus to turn on the suicidal NETosis mode with ROS production.

Since JNK modulates apoptosis^18, 19, 51^, it is pertinent to ask whether JNK inhibition promotes apoptosis during the suppression of LPS-mediated NETosis. In apoptotic cells, caspase-3 is cleaved and DNA is condensed, while in NETotic cells, caspase-3 is not cleaved and DNA is decondensed^52, 53^. Confocal microscopy results indicate that the media control, with and without JNK inhibitor, results in the same amount of cleaved caspase-3 and DNA condensation as does the FasL-positive control (Fig. 7; Supplementary Fig. 7). JNK inhibition significantly inhibited NETosis in LPS-treated cells, and also reduced the rate of apoptosis. Thus, JNK inhibition suppresses death of neutrophils even when the cells were treated with high concentrations of LPS (25 μg/ml). JNK plays a role in pyocyanin or crystal-mediated NETosis-like cell death^54, 55^. However, the molecular mechanisms of pyocyanin and crystal-mediated neutrophil death remain unknown. In this study, we have delineated potential mechanisms, and also studied the involvement of JNK in NETosis induced by increasing MOI of Gram-negative bacteria (Fig. 8). Our previous studies also show that increasing MOI of *E. coli* and *P. aeruginosa* increases Nox-dependent suicidal NETosis^5^. Similar to LPS-mediated NETosis, JNK inhibition suppresses *E. coli*- and *P. aeruginosa-*induced NETosis indicating that Gram-negative bacteria also follow similar pathways for regulating NETosis.

**Figure 9:**
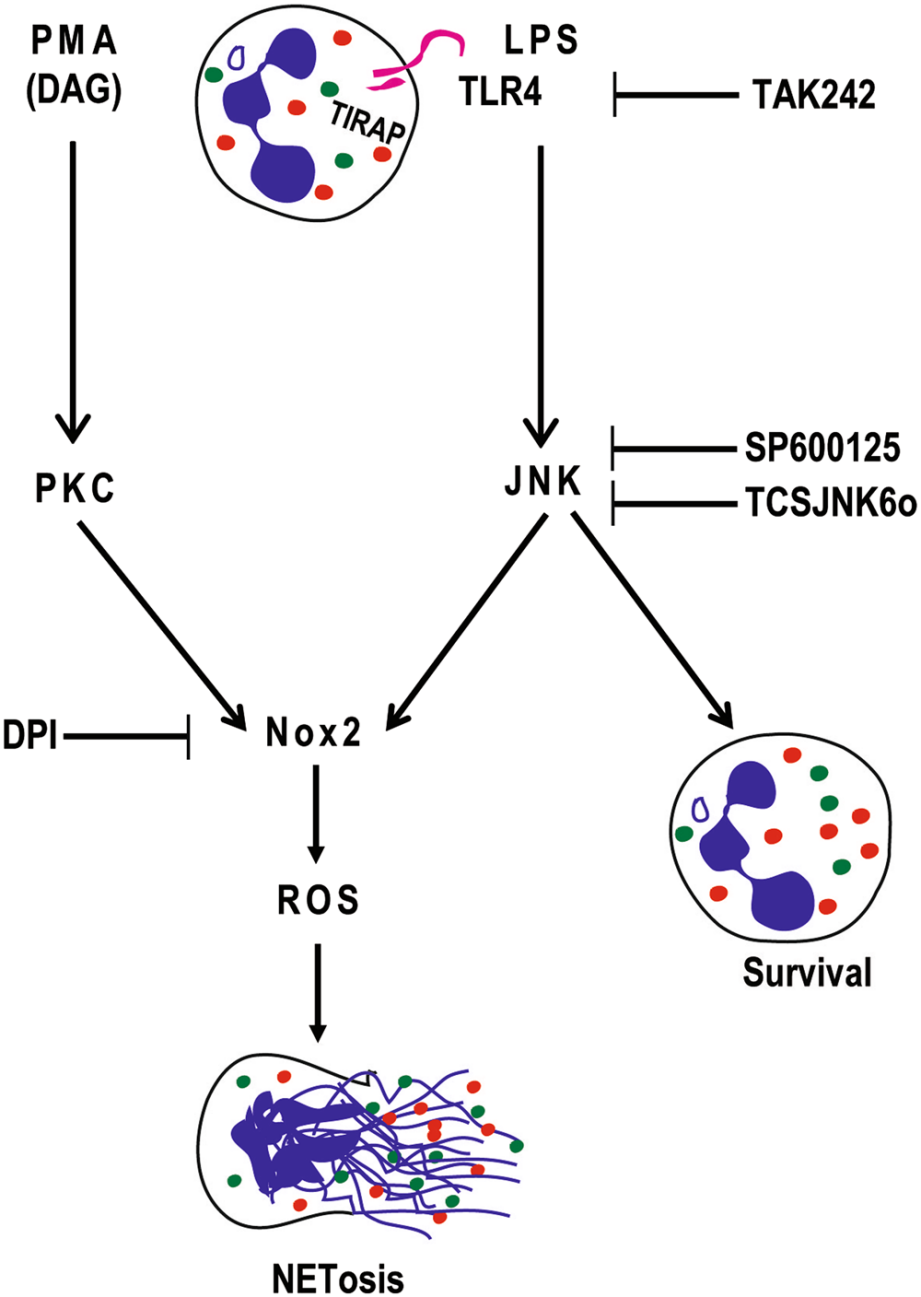
LPS-TLR4-JNK axis regulates Nox-dependent suicidal NETosis. PMA and LPS regulate ROS production in neutrophils via PKC and JNK, respectively. Blocking TLR4 signaling with TAK242 and JNK activation with SP600125 or TCSJNK6o during LPS-mediated NETosis results in the suppression of ROS production and Nox-dependent NETosis, and increased survival of neutrophils. Nox inhibitor DPI suppresses both PMA- and LPS-mediated NETosis. Color dots represent different granular proteins. Granule decorated strings represent DNA and NETs. Specific inhibitors and their points of inhibitions are indicated. Based on all the data obtained in this study, we propose that LPS-TLR4-JNK signaling cascade acts as a sensor of LPS concentrations and bacterial load, and turns on the neutrophil death mode to the suicidal Nox-dependent NETosis.

In conclusion, we have identified a crucial new role for JNK in regulating LPS- and Gram-negative bacteria-mediated, Nox-dependent NETosis. JNK is essential for endotoxin concentration-dependent LPS-TLR4-mediated NETosis to regulate ROS production, which is necessary for NETosis. We also show that JNK inhibition does not lead to apoptosis or other forms of death, but delays cell death resulting from LPS treatment. Furthermore, this study identified a fundamental difference between PMA- and LPS-mediated Nox-dependent NETosis. Additionally, we demonstrated that similar to LPS, Gram-negative bacteria also require JNK to induce NETs. These findings now place JNK as an important regulatory MAPK required for directly inducing LPS:TLR4:neutrophil-mediated Nox-dependent suicidal NETosis and regulating neutrophil survival (Fig. 9). Activation of JNK acts as a molecular rheostat that senses LPS concentration and bacterial load to turn on Nox-dependent suicidal NETosis. The LPS-TLR4-JNK activation axis determines the viability and fate of these neutrophils: to be or not to be NETotic neutrophils.

## Materials and Methods

### Primary Human Neutrophils

The study protocol was approved by the Hospital for Sick Children ethics committee. All methods were performed in accordance with the relevant guidelines and regulations. Neutrophils from healthy male donors were used in the study. Donors with eosinophils, or either too low or too high neutrophil count were excluded from the study. Typically, our isolation procedure yields 1–2 million neutrophils per ml of blood. Blood from donors that fall outside of this range has not been used in the study. We collected peripheral blood from healthy male donors in K2 EDTA blood collection tubes (Becton, Dickinson and Co.) after obtaining informed consent. Neutrophils were isolated from these blood samples using the PolymorphPrep (Axis-Shield) protocol. During neutrophil isolation, red blood cells were lysed with a 0.2% (w/v) NaCl hypotonic solution for 30 seconds followed by adding an equal volume of 1.6% (w/v) NaCl solution with 20 mM HEPES buffer to obtain the isotonic condition. Two more washes of 0.85% (w/v) NaCl with 10 mM HEPES solution was done to eliminate red blood cell debris. Neutrophils were resuspended in RPMI medium (Invitrogen) containing 10 mM HEPES (pH 7.2), and counted and viability checked by trypan blue assay before using them for the experiments. Cell density of purified neutrophils was quantified using a hemocytometer, and the purity of the neutrophils was determined by Cytospin preparations. Neutrophil preparations with >95–98% were used in all the experiments, reported in this study. NETs were induced in cells by the addition of 25 nM PMA, and 25 µg/ml LPS (*Escherichia coli* 0111:B4). For inhibitory assays, the cells were incubated in different concentrations of JNK inhibitor SP600125 or TCSJNK6o, and TLR4-TIRAP/TRAM blocker TAK242 for 30–60 minutes before inducing NETosis. Each condition had a technical duplicate. Each assay was repeated with different donors to obtain biological replicates (n = 3–5; see specific details in figure legends).

### Sytox Green NETosis Assay

For measuring NETosis kinetics, the Sytox Green cell-impermeable DNA dye (Life Technologies) was used at a concentration of 5 µM. The fluorescence of the dye was measured with the POLARstar OMEGA fluorescence microplate reader (BMG Labtech) at 30-minute time intervals for up to 240 minutes after cell activation. To calculate % NETosis in each condition, the green fluorescence at time 0-min was subtracted from the fluorescence at each time point and was then divided by the fluorescence values of cell lysed with 0.5% (v/v) Triton X-100 (representing total DNA). The DNA release in each condition is presented as percentages DNA release of total DNA. We ensured that the spontaneous background activation in these samples is less than 30% (of total DNA, by Sytox green assay) to minimize variability. Assays were not completed if the neutrophils were activated at baseline. Neutrophils from the donors used in this study responded well to PMA, and showed 65–100% of NETosis (of total DNA, by Sytox green assay).

### DHR123-based ROS Detection Assay

Intracellular ROS were measured using the DHR123 dye (Life Technologies). The cells were incubated with 25 μM DHR123 for 10 min. After washing, the DHR123 preloaded cells were seeded into 96-well plate and activated with negative control (only media), PMA and LPS, with and without prior incubation of JNK inhibitors or TLR4 inhibitor. The fluorescence was measured every 10 minutes up to 40 minutes by an Omega fluorescence microplate reader. Plate reader assay was used for quantifying ROS, while for the visualization and qualitative analyses confocal imaging was performed in cells under above mentioned different experimental conditions.

### Bacterial Culture

*Escherichia coli* (Y1088) and *Pseudomonas aeruginosa* (mPA01) were grown overnight in sterile LB-broth, selected from single colonies in LB-agar plates. Prior to neutrophil activation (NETosis induction), bacterial cultures were diluted by a factor of ten and sub-cultured for 3 hours. Actively growing cells were harvested and washed three times in 5 mL of phosphate-buffered saline (PBS, pH 7.4) by centrifugation at 5000 × g for 5 minutes at 4 °C. After the final resuspension, the concentration of the optical density (OD) was determined at 600 nm, and different multiplicity of infection (MOI) were calculated using the colony forming unit (CFU) using a formula established in the lab by empirical methods; [CFU] × 10^8^ = (OD_600_) × 30.88 − 99,607.

### Immunoblot Analysis

For the immunoblot analysis, 1 × 10^6^ cells in each experimental condition were activated, incubated at 37 °C for indicated time points and then placed on ice after the incubation time. The cells were then centrifuged at 20,000 rcf at 4 °C for 10 min. The supernatant was then discarded and the cell pellets were lysed using the lysis buffer containing 1% (w/v) Triton X-100, 25 mM NaF, 50 mM Tris, 10 mM KCl, 10 µg/ml Aprotinin, 2 mM PMSF, 1 mM Levanisole, 1 mM NaVO_3_, 0.5 µM EDTA, 25 µM Leupeptin, 25 µM Pepstatin, 1 protease inhibitor cocktail tablets per 5 ml (Roche), and 1 phosphatase inhibitor cocktail tablet per 10 ml (Roche). The samples were then vortexed for 10 seconds followed by three times of sonication using an aquasonic sonicator (VWR, model 50D at the highest power setting), at 8–10 °C, 3-min each). A quarter (represents 4x) volume of 5 × loading dye (125 mM Tris.HCl at pH 6.8, 6% (w/v) SDS, 8% (v/v) β-mercaptoethanol, 18% (v/v) glycerol, 5 mM EDTA, 5 mM EGTA, 10 µg/ml Leupeptin, 10 µg/ml Pepstatin, 10 µg/ml Aprotinin, 10 mM NaF, 5 mM NaVO_3_, and 1 mM Levamisole) was added followed by 10 min of incubation at 95 °C with 350 rcm shaking. The samples were size-fractionated in a 5% stacking and 10% resolving gel at 100 V and transferred on a nitrocellulose membrane for 90 minutes at 400 mA. After transfer, the membranes were blocked with 5% (w/v) milk or BSA (for p-JNK immunoblots) in 0.05% PBST for 1 hour at room temperature. The membranes were incubated in the primary antibody at 4 °C overnight followed by 3 washes with 0.1% PBST for 30 minutes. The antibodies used were: anti-phospho-SAPK/JNK (Thr183/Tyr185) (81E11, Cell Signaling) rabbit mAb at 1:1000; anti-cleaved caspase-3 (Asp175, Cell Signaling) rabbit at 1:1000; anti-JNK1 (2C6, 3808, Cell Signaling) mouse mAb at 1:1000; anti-JNK2 (56G8, 9258, Cell Signaling) rabbit mAb at 1:1000; and anti-GAPDH (FL-335, Santa Cruz) rabbit pAb at 1:2500. The membranes were then incubated in the secondary antibody solution for 1 hour and then washed 3 times with 0.1% PBST for 30 minutes. The secondary antibodies used were: Donkey anti-rabbit IgG-HRP (31458, Thermo Fisher) at 1:7500; and goat anti-mouse IgG-HRP (ab6789, Abcam) at 1:7500. The densitometry analysis of the blots was done using the Image Studio software (LI-COR Biotechnology). Cleaved caspase-3 and phospho-JNK blots were normalized to the GAPDH.

### Confocal Microscopy

For confocal imaging, 150 μL of 150,000 cells were used in an 8-chamber slide (BD Falcon). After various incubation time, the cells were fixed with 4% (w/v) PFA overnight in 4 °C. The samples were then washed three times with PBS and then permeabilized with 0.05% (w/v) Triton X-100. After washing three more times with PBS, the cells were blocked with 5% BSA solution for 1 hour. The samples were then incubated in primary antibodies for 2 hours followed by 3 washes with PBS. The antibodies used are: anti-phospho-SAPK/JNK (Thr183/Tyr185) (81E11, Cell Signaling) rabbit antibody at 1:1000; anti-myeloperoxidase mouse antibody (ab25989, Abcam) at 1:1000; and anti-cleaved caspase-3 rabbit antibody (Asp175, Cell Signaling) at 1:1000 were used to stain p-JNK, MPO and cleaved caspase-3, respectively. The samples were then treated with the secondary antibodies and nucleic acid-binding DAPI dye (Invitrogen) at 1:100 for 2 hours. The secondary antibodies used were: Donkey anti-mouse (488 nm, ab98766, Abcam) at 1:1000; and goat anti-rabbit (Alexa Fluor 555, A21428, life technologies) at 1:1000. The images were then taken using an Olympus IX81 inverted fluorescence microscope with a Hamamatsu C9100-13 back-thinned EM-CCD camera and Yokogawa CSU × 1 spinning disk confocal scan head with Spectral Aurora Borealis upgrade, 4 separate diode-pumped solid state laser lines (Spectral Applied Research, 405 nm, 491 nm, 561 nm, and 642 nm). The images were taken at 40 ×/0.95 magnification and then analyzed with Volocity Software (Perkin-Elmer).

### Statistical Analysis

All the statistical analyses were performed on GraphPad Prism 7. One-Way ANOVA with Dunnett and Tukey’s post tests, Two-way ANOVA with Bonferroni post-test or t-test was done as appropriate. A p-value of <0.05 was considered to represent significant differences between conditions and all data are presented as mean ± SEM.

## Electronic supplementary material

Supplementary Figures
