# Supplementary material for: JNK Activation Turns on LPS- and Gram-Negative Bacteria-Induced NADPH Oxidase-Dependent Suicidal NETosis
Source: Sci Rep. 2017 Jun 13;7:3409. doi: 10.1038/s41598-017-03257-z (PMC5469795; doi:10.1038/s41598-017-03257-z)
Supplement: Supplementary file 1 — Supplementary Figures [file 41598_2017_3257_MOESM1_ESM.pdf]

Supplementary Figures

JNK Activation Turns on LPS- and Gram-Negative Bacteria-Induced NADPH Oxidase-Dependent Suicidal NETosis

Meraj A. Khan[1,2,#], Armin Farahvash[1,2,#], David Douda[1,2], Johann-Christoph Licht[1], Hartmut Grasemann[1,3,4], Neil Sweezey[1,3] & Nades Palaniyar[1,2,4,*]

[1]Program in Translational Medicine, Peter Gilgan Centre for Research and Learning, The Hospital for Sick Children, Toronto. [2]Department of Laboratory Medicine and Pathobiology, [3]Departments of Paediatrics and Physiology, and [4]Institute of Medical Sciences, Faculty of Medicine, University of Toronto.

[#], both of these authors contributed equally.

**Address correspondence to: Program in Translational Medicine, Peter Gilgan Centre for Research and Learning, The Hospital for Sick Children, 686, Bay St., Toronto, ON, Canada M5G0A4.* nades.palaniyar@sickkids.ca

Running title: *LPS:TLR4:JNK axis determines LPS-mediated NETosis*

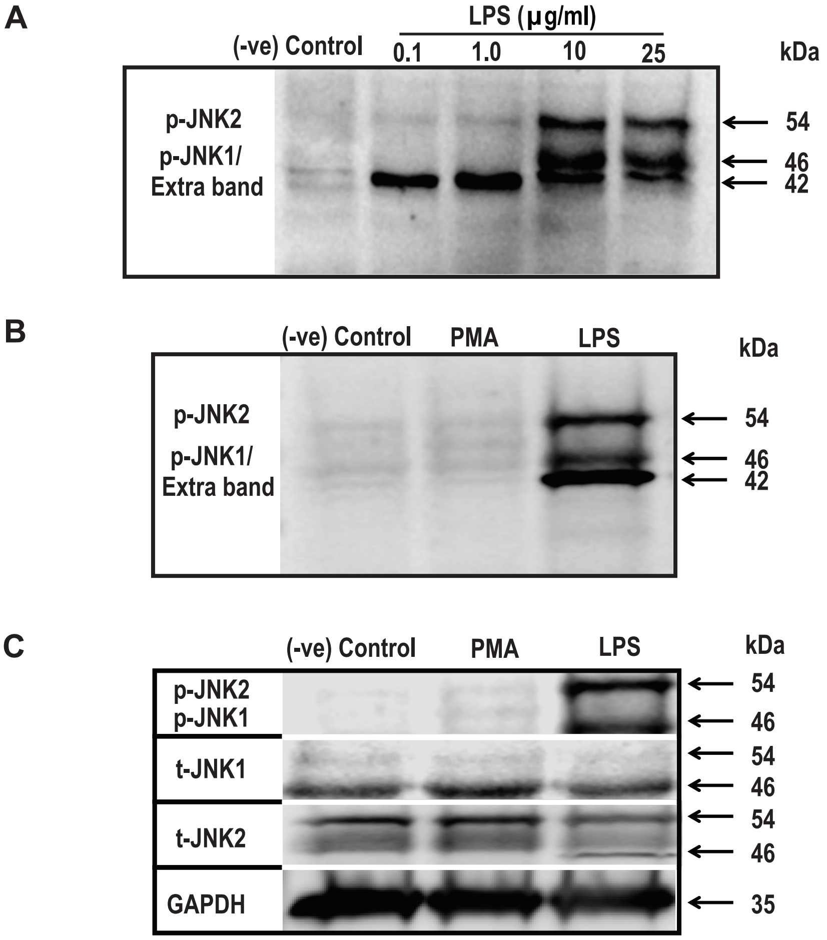

Figure S1. Total JNK1 and JNK2 levels do not change within the 30-minute incubation period. (A) Full Western blot of Figure 1A. **(B)** Full Western blot of Figure 1C. **(C)** Total JNK and GAPDH. Human neutrophils were lysed after 30 minutes of stimulation with –ve control (media only), PMA (25 nM) or LPS (25 μg/ml). Immunoblots show that LPS, but not PMA activates JNK in neutrophils. Total JNK1 (t-JNK1) and JNK2 (t-JNK2) were detected with different antibodies, and blotted separately. Antibodies to p-JNK detect both p-JNK1 and p-JNK2. GAPDH blots were used as loading controls (n = 3). In some blots, an extra band at the location of p-JNK1 was detected, and its importance is unknown (**A, B**). Overall, total JNK expression is similar among different experimental conditions within the 30-minute experimental period. Therefore, p-JNK levels represent activation, and not new protein synthesis.

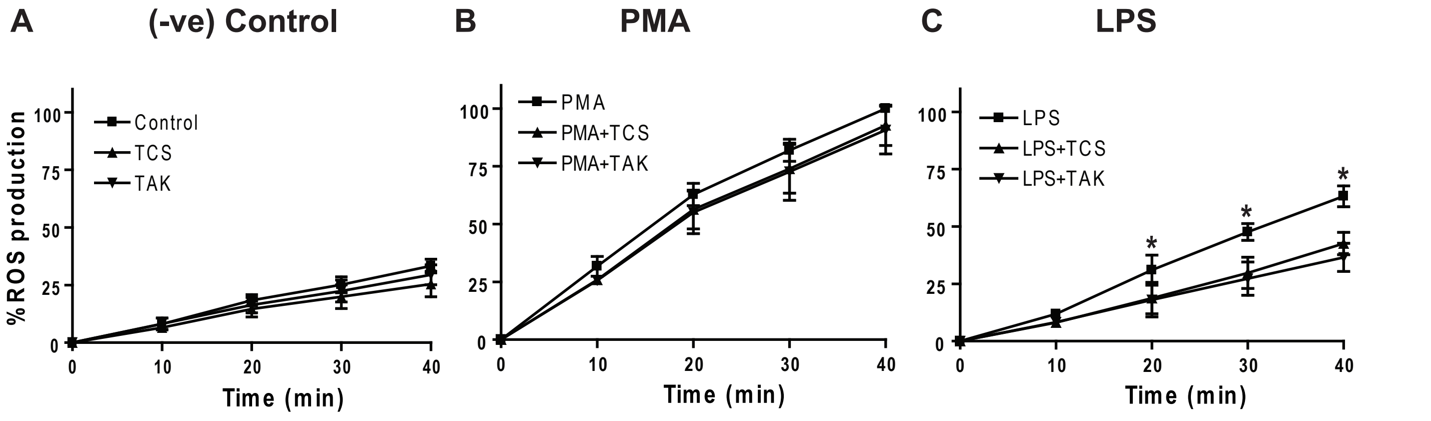

Figure S2. Inhibitors of JNK activation TCS (TCSJNK6o) and TLR4 signaling TAK (TAK242) suppresses ROS production in LPS treated neutrophils. (A-C) ROS inhibition kinetics was determined by R123 intensities (negative control, PMA, and LPS; n=3; *p value <0.05; Two-way ANOVA with Bonferroni's post test conducted at each time point).

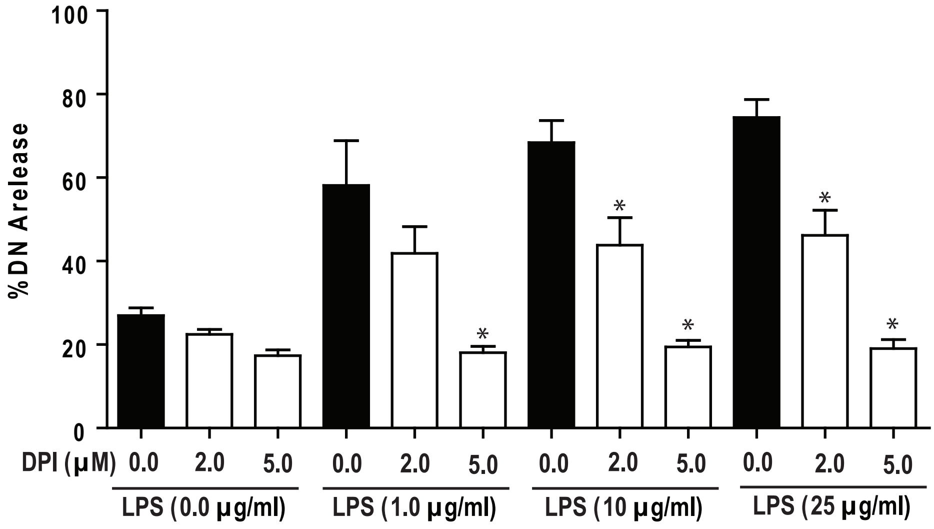

Figure S3. Nox inhibitor DPI suppresses LPS-mediated NETosis. Neutrophils were treated with different concentrations of DPI (2 and 5 µM) and activated with different dosage of LPS (0, 1, 10, 25 µg/ml). The Sytox Green fluorescence (%DNA release) shows the dose-dependent suppression of DPI in LPS-mediated NETosis (n=3; *, $p<0.05$; One-way ANOVA with Dunnett post test conducted between no DPI and 2.0, 5.0 µM DPI, in each LPS condition).

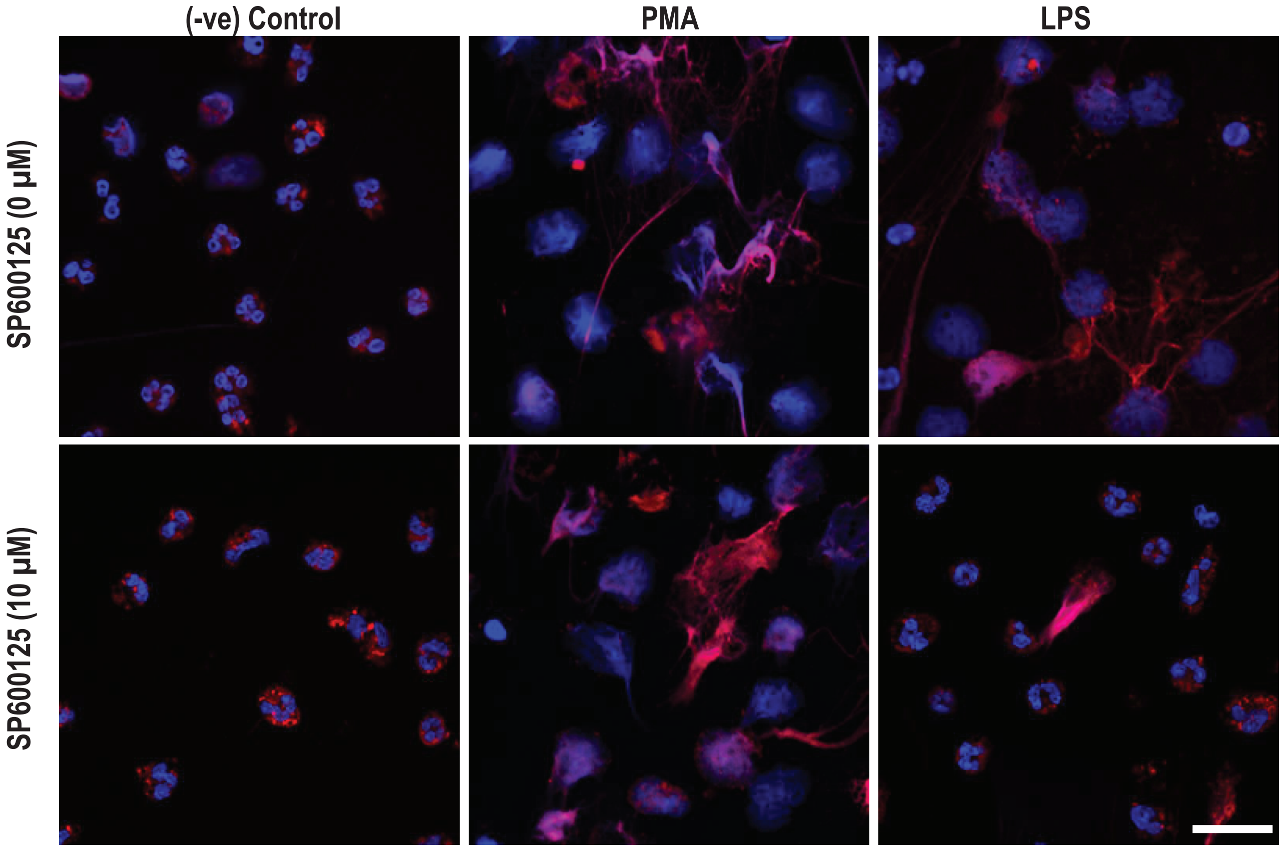

Figure S4. Confocal microscopy images at low magnification show that JNK inhibitor SP600125 suppresses LPS-mediated NETosis (Blue, DAPI staining for DNA; Red, MPO; n = 3; scale bar 20 µm; See magnified images in **Fig. 4**).

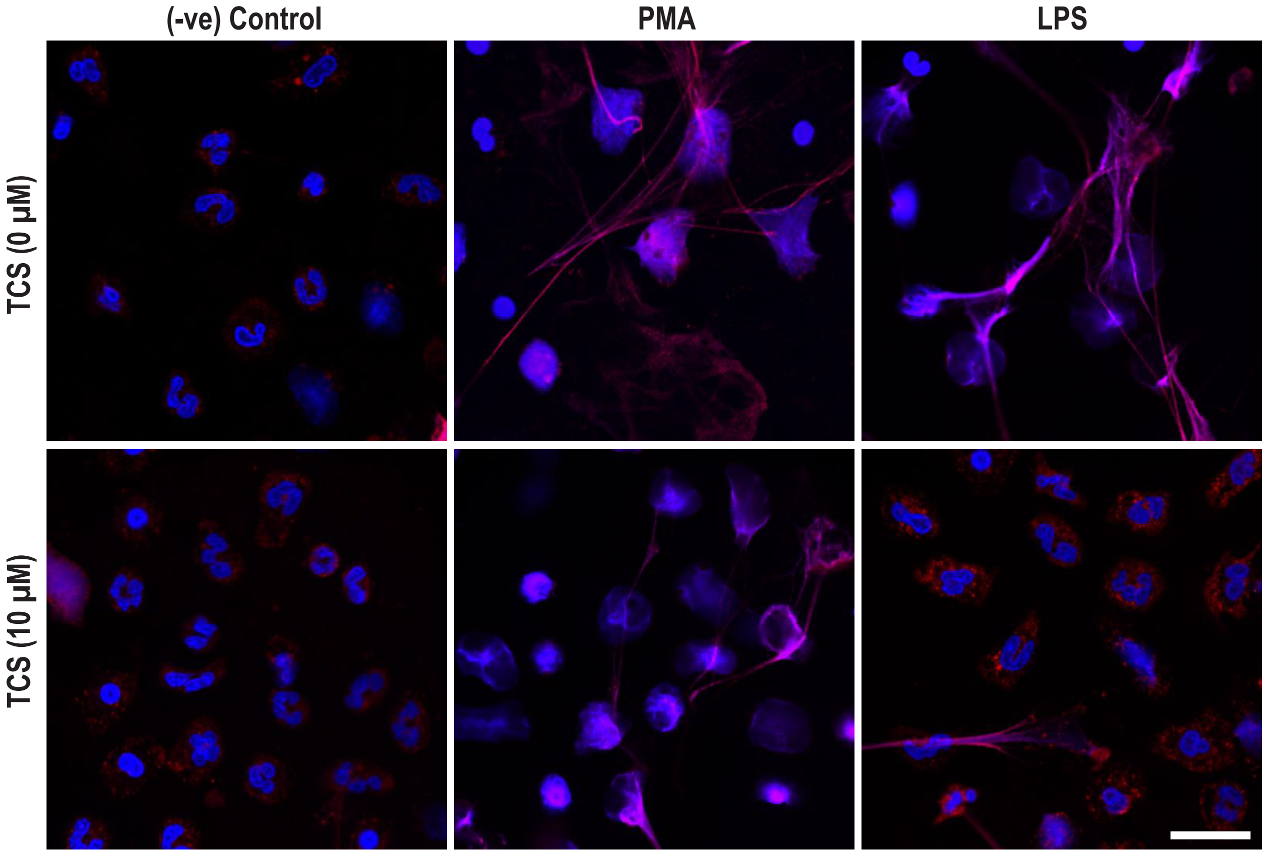

Figure S5. Confocal microscopy images at low magnification show that JNK inhibitor TCSJNK6o suppresses LPS-mediated NETosis (Blue, DAPI staining for DNA; Red, MPO; n = 3; scale bar 20 μm; See magnified images in **Fig. 5**).

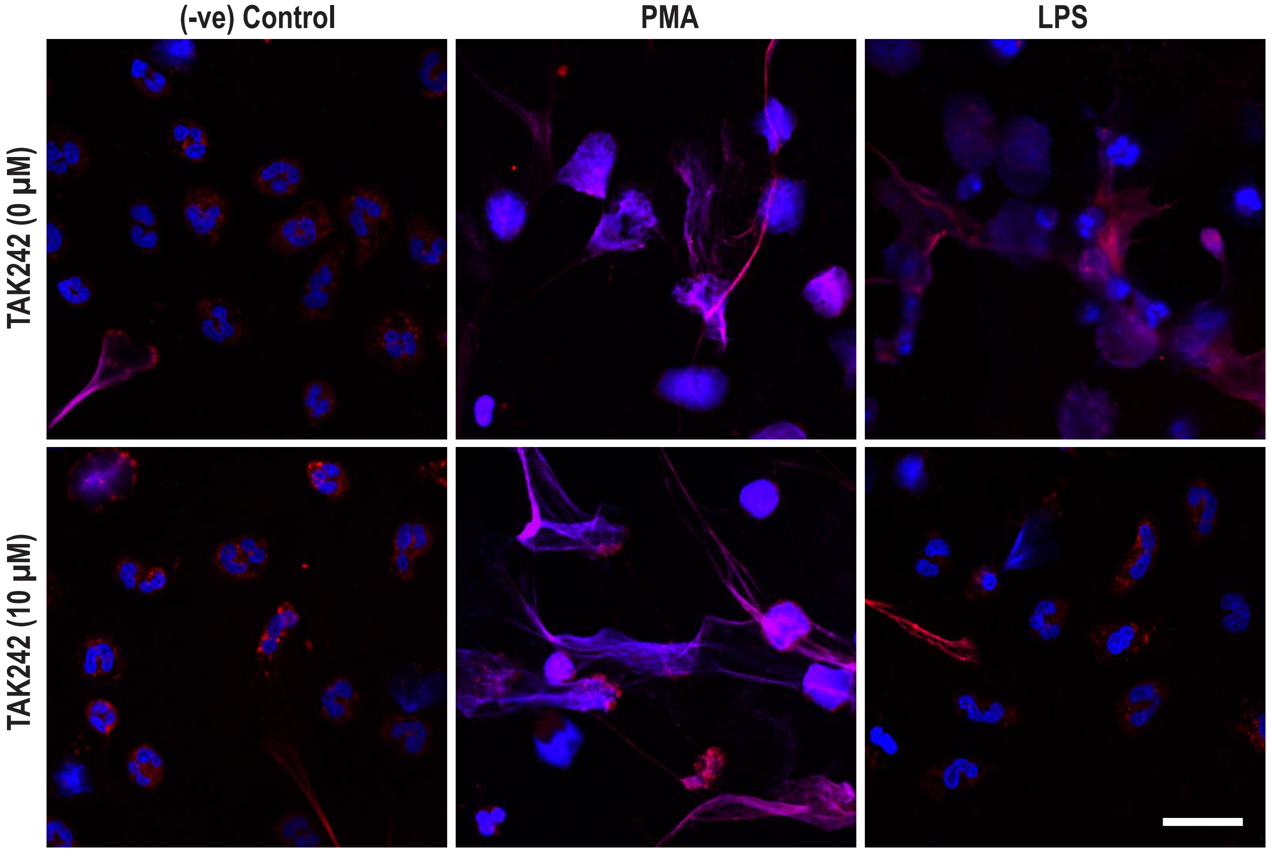

Figure S6. Confocal microscopy images at low magnification show that TLR4 inhibitor TAK242 suppresses LPS-mediated NETosis (Blue, DAPI staining for DNA; Red, MPO; n = 3; scale bar 20 μm; See magnified images in **Fig. 6**).

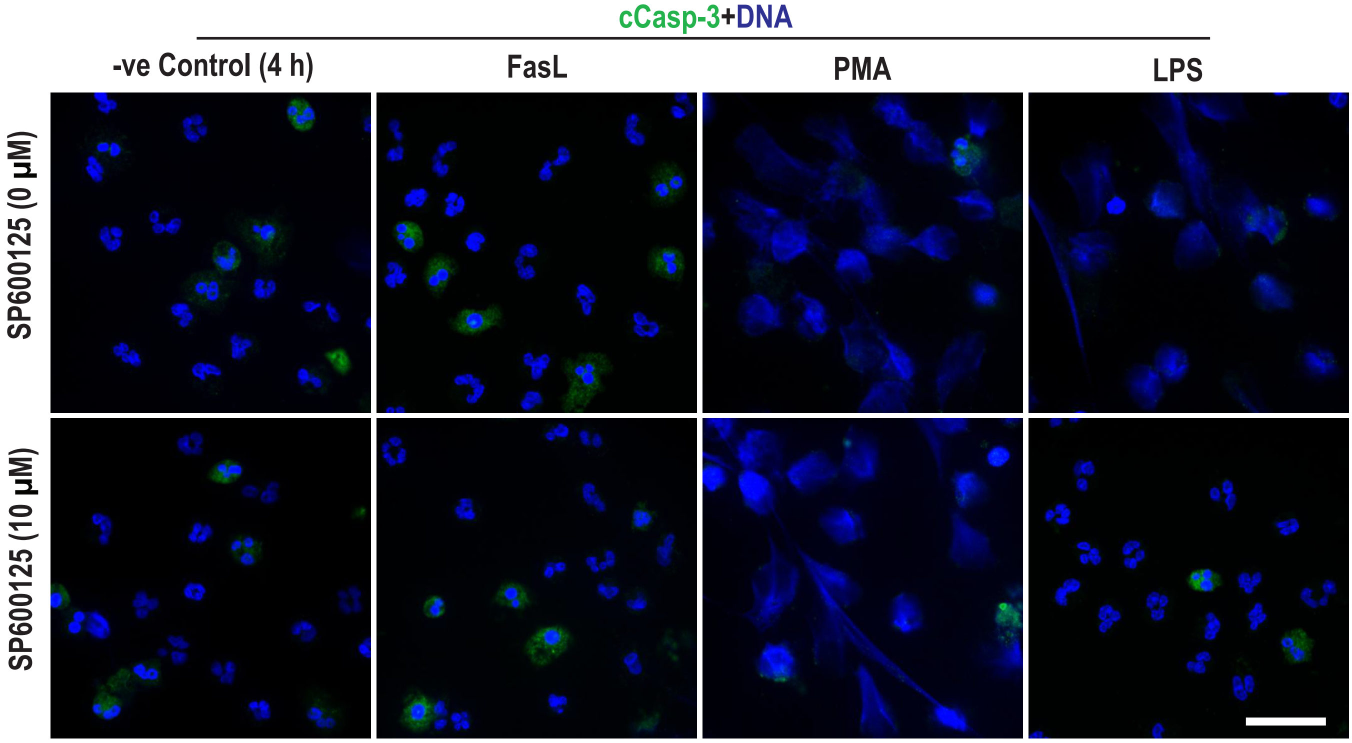

Figure S7. Confocal microscopy images at low magnification show that JNK inhibition by SP600125 does not induce other forms of cell death in LPS-treated neutrophils, at 4-hour time point (Blue, DAPI staining for DNA; Green, cCasp-3 indicating apoptosis; n = 3; scale bar 20 μm; See magnified images in **Fig. 7C**).
